# Electrochemical and Optical Sensors for Real-Time Detection of Nitrate in Water

**DOI:** 10.3390/s23167099

**Published:** 2023-08-11

**Authors:** Kartikay Lal, Swapna A. Jaywant, Khalid Mahmood Arif

**Affiliations:** Department of Mechanical and Electrical Engineering, SF&AT, Massey University, Auckland 0632, New Zealand; k.lal@massey.ac.nz (K.L.); s.jaywant@massey.ac.nz (S.A.J.)

**Keywords:** electrochemical sensing, nitrate, optical sensing, real-time sensing, wireless sensor networks

## Abstract

The health and integrity of our water sources are vital for the existence of all forms of life. However, with the growth in population and anthropogenic activities, the quality of water is being impacted globally, particularly due to a widespread problem of nitrate contamination that poses numerous health risks. To address this issue, investigations into various detection methods for the development of in situ real-time monitoring devices have attracted the attention of many researchers. Among the most prominent detection methods are chromatography, colorimetry, electrochemistry, and spectroscopy. While all these methods have their pros and cons, electrochemical and optical methods have emerged as robust and efficient techniques that offer cost-effective, accurate, sensitive, and reliable measurements. This review provides an overview of techniques that are ideal for field-deployable nitrate sensing applications, with an emphasis on electrochemical and optical detection methods. It discusses the underlying principles, recent advances, and various measurement techniques. Additionally, the review explores the current developments in real-time nitrate sensors and discusses the challenges of real-time implementation.

## 1. Introduction

Water pollution is a global issue that demands continuous monitoring to ensure the safety and integrity of our water sources. The presence of nitrate in water can be the result of various sources such as runoff or seepage from fertilized soil, wastewater, landfills, animals, or urban drainage. This phenomenon is known as eutrophication and poses a significant concern for estuaries. Eutrophication refers to the enrichment of the environment with nutrients, leading to the excessive growth of plants and algae in estuaries and coastal waters [1]. Substances such as heavy metals, nutrients, and pathogens are considered to be primary contaminants [2]. Nitrogen is the most commonly available nutrient in the atmosphere. Different gaseous forms of nitrogen are present in the air, such as nitrogen (N_2_), nitrous oxide (N_2_O), nitric oxide (NO), nitrogen dioxide (NO_2_), and ammonia (NH_3_). These gases react with rainwater and produce nitrate. This nitrate mixes with groundwater or becomes a part of the soil layer. On the other hand, many anthropogenic activities, such as the excessive usage of fertilizers in agriculture and the disposal of animal excretion and industrial waste, lead to an excessive amount of nitrates in the atmosphere and are the primary cause for nitrate pollution in water [3].

**Figure 1:**
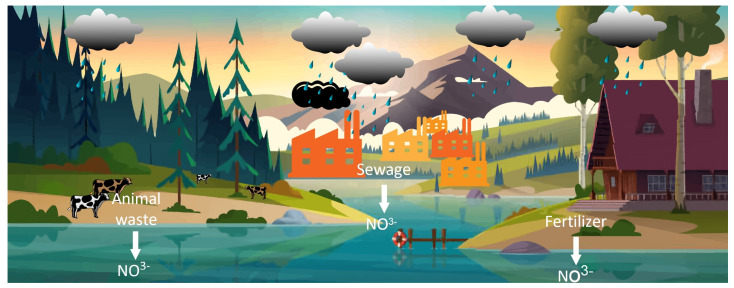
Nitrate leaching from soil/fields and industrial waste into surface water and groundwater.

Nitrate ions affect nature significantly. The presence of nitrate ions can have a significant impact on the environment [4]. When present in nominal amounts, they can promote aquatic life. However, an excessive amount of nitrate could lead to harmful effects on the aquatic ecosystem, such as the increased production of algae and phytoplankton, stimulating eutrophication, as illustrated in Figure 1. As these organisms decompose, they utilize more oxygen, leading to adverse effects on marine life. Similarly, nitrate poisoning can also occur in animal farming, resulting in livestock abortions and reduced dairy production, which lead to significant losses for farmers [5]. On the other hand, nitrate consumption can be beneficial to humans, as it can improve blood flow and lower blood pressure [6]. Meanwhile, excessive nitrate intake is always hazardous for humans and animals. Nitrate can be used as a food preservative in the food processing industry, and it often helps in curing meat; hence, it is mostly used in fish and salted meat preparation.

Furthermore, bacteria found in the human system can reduce nitrate ions to nitrite, resulting in the formation of carcinogenic N-nitrosamine compounds. As a consequence of these compounds, many health issues can occur, including esophageal and gastric cancer [7], spontaneous abortion, congenital disabilities [8], and Parkinson’s disease [5]. Newborns can experience “blue baby syndrome” or methemoglobinemia. Thus, nitrate has a vital role in environmental and human health monitoring, and its detection and estimation are important [3,9]. Hence, authorities such as European Union Directives (EUDs) [6,10] and the World Health Organization (WHO) [6,11] suggest 50 mg/L as the maximum permissible nitrate concentration in drinking water and food. Italian regulations recommend 45 mg/L for adults and 10 mg/L for infants [6,10]. The United States Environmental Protection Agency (EPA) advises 44.2 mg/L [6,12]. Additionally, the EPA, EUDs, and Australia and New Zealand Food Standards indicate 365, 10–500, and 50–500 mg/kg as the suggested levels in food, respectively [6,13].

Traditionally, a variety of analytical techniques have been utilized for nitrate detection, including atomic absorption spectrometry, inductively coupled plasma mass spectrometry, ion chromatography, UV/VIS spectrometry, chemiluminescence, and electrophoresis. These techniques have demonstrated a high degree of nitrate detection accuracy. However, such techniques require the periodic collection of water samples that would need to be transported to a laboratory for testing. This process could be costly due to the need for a specialized facility with expensive laboratory equipment, chemical reagents, and skilled technical personnel to operate the equipment. Such methods can generate chemical waste, adding to the overall cost. Moreover, periodic sample collection may not capture short-term contamination events [14,15]. For this reason, it is always desirable to have a simple and field-deployable device that can perform real-time nitrate detection cost-effectively. Hence, an increase in available portable monitoring devices has been observed. While implementing these portable devices, researchers come across various challenges, such as rapidly changing environmental and temperature conditions, limitations in accessing and servicing the equipment, and a lack of grid-power connections [16]. An ideal field-deployed nitrate sensor for water must be sensitive enough to detect nitrate concentration lower than 1 mg/L in surface water. However, there are several challenges involved with the detection of nitrate in real-world samples. The complexity of the sample matrix is the most prevalent issue. Real-world samples often contain various impurities, such as dissolved solids, organic matter, and other ionic content, which interfere with the detection of nitrate and could affect measurement accuracy. Another challenge for a field-deployable sensor is biofouling. Over a period of time, biological growth on the sensor can interfere with the accurate detection of nitrate, which could require the regular cleaning and maintenance of the sensor.

In recent years, various methods have been explored to develop real-time nitrate monitoring systems that incorporate analytical techniques such as chromatography, colorimetry, electrochemistry, and spectroscopy. Among these methods, electrochemical analysis has emerged as a promising approach due to its simplicity, high sensitivity, accuracy, wide measuring range, affordability, user-friendliness, and suitability for field-deployable applications. As a result, researchers have begun to view electrochemical sensing as a favorable alternative to the more expensive conventional analytical techniques [14]. Hence, this review aimed to provide an overview of the detection techniques that can be employed for the real-time detection of nitrate ions in water with an ideal accuracy of less than 1 mg/L, which would allow integration with field-deployable applications. The electrochemical approach is thoroughly investigated, including its underlying principles, electrode configuration, and various measurement strategies. The optical methods are discussed in detail as well. Furthermore, the review provides a thorough examination of current real-time nitrate sensors and their recent improvements. Lastly, the advantages and disadvantages of the potential methods are briefly highlighted.

## 2. Electrochemical Methods

### 2.1. Working Principle

**Figure 2:**
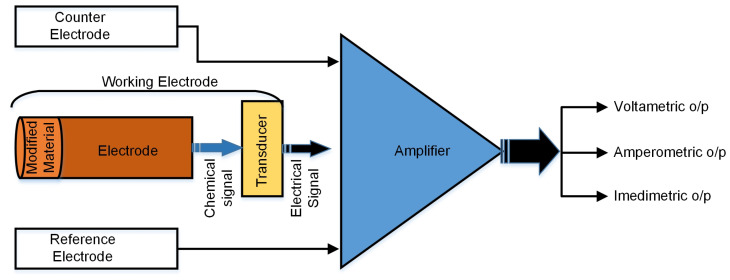
Working principle of the three-electrode electrochemical method.

In the electrochemical methods, oxidation or reduction are used to produce a current to detect a particular analyte [17]. The primary element of any electrochemical sensor is an electrochemical transducer. A transducer consists of a sensing element that reacts with the target analyte and transforms the chemical reaction into an analytical signal. Typically, the electrochemical sensor is a three-electrode system that includes a reference electrode (RE), sensing/working electrode (WE), and counter electrode (CE). The electrolyte reaction occurs at the working electrode. This electrode is generally modified with a nanomaterial to accelerate the electrolytic reaction. The reference electrode allows the correct application of the working electrode potential. The counter electrode is used to complete the circuit and helps continue the electron flow [6]. Figure 2 illustrates the working principle of the method in a three-electrode system.

**Figure 3:**
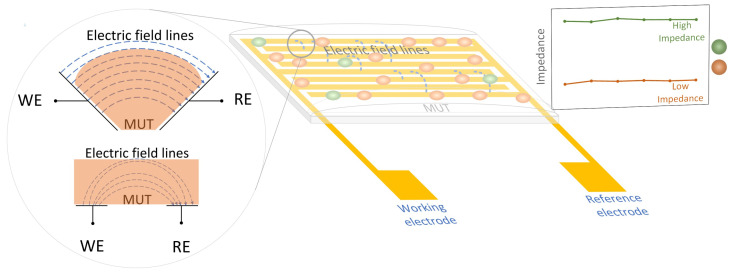
Working principle of 2D planar electrodes.

On the other hand, simpler “two-dimensional planar electrodes” refer to a parallel configuration of electrodes that are constructed on a flexible or rigid substrate. A traditional pair of electrodes comprises a working electrode (WE) and a reference electrode (RE) [18]. The RE is used as the excitation electrode, and the WE senses the resulting phase shift of the current with respect to the input signal on the RE. The capacity for single-sided access to the MUT makes IDEs ideal for in situ analysis. Figure 3 illustrates the working principle of 2D planar electrodes. Recently, 2D planar-style sensors have gained popularity in sensing applications. The most commonly employed pattern is a parallel comb-like structure, referred to as an interdigitated geometrical pattern. However, there are other geometric patterns that can be used in sensing applications, whose performances were evaluated in our previous work [19]. The working principle of planar electrodes involves the generation of an electric field when a low-amplitude AC signal is applied across two electrodes. The ions present in the material under test (MUT) react to the applied signal by aligning themselves with the produced electric field, surpassing their random thermal motion and leading to modifications in parameters like electrical impedance [20]. This interaction results in changes in the electric field and the current in the working electrode. Either of the two well-known metrics, namely capacitance and impedance, can be utilized to determine the ionic content in the solution [21]. Table 1 highlights some advantages and disadvantages of two- and three-electrode systems.

### 2.2. Electrode Design

Nitrate reduction on the cathode surface is an essential characteristic of electrochemical sensors. Hence, the electrode material and electrode design play a significant role in the performance of an electrochemical sensor. In the last two decades, metals like Ru, Rh, Ir, Pt, Pd, Cu, Ni, and Ag have been studied for nitrate detection using electrochemical analysis. Cu was found to be the most promising electrocatalyst for the nitrate reduction reaction among all of these [22,23,24,25,26]. Hence, many Cu-based electrodes, such as copper-deposited platinum microelectrodes [27], copper-coated bismuth [28], copper-modified pencil graphite [29], an array of copper microelectrodes [30], copper co-modified carbon fiber electrodes [31], and nanowire-based Cu electrodes [22], have been used in nitrate analysis.

**Figure 4:**
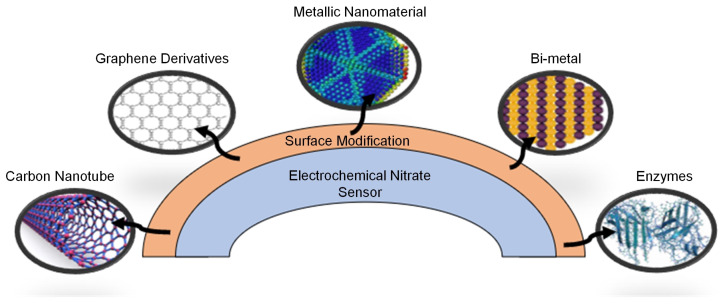
Materials used for the surface modification of the working electrode.

Furthermore, various catalyst nanomaterials have been synthesized for the working electrode to achieve significant results in nitrate detection. Modified electrodes in electrochemical analysis lead to advantages like a rapid response, decreased overpotential, high sensitivity, and high selectivity compared to conventional electrochemical sensors. The nanomaterials (Figure 4) that have been considerably studied for the surface modification of working electrodes in nitrate sensors include graphene derivatives [32,33,34]; carbon nanotubes (CNTs) [24,35], carbon fibers [36]; metal, bi-metal, and metal oxide nanoparticles; and conductive polymers. Alongside these, enzymes have been explored for the same purpose [6]. Various adaptations of electrode materials have been employed in conjunction with IDEs. These electrodes are fabricated from metals like gold [37], silver [38], copper [39], and carbon [31], which exhibit excellent electrical conductivity. For example, IDEs have frequently been utilized to measure the pH level of aqueous solutions by detecting H+ ions [37,40,41]. Electrodes are often modified for the enhanced detection of the desired ion [42]. Modifications with nanoparticles such as gold, silver, and non-metallic elements like sulphur and carbon have also been applied [26,43,44,45]. Similarly, for the detection of nitrate ions in water, electrodes are often modified with various metals for enhanced sensitivity to nitrate ions [46,47].

### 2.3. Electrochemical Measurement Techniques

The sensor’s output can be either current, voltage, or impedance. Hence, electrochemical methods for nitrate detection can be categorized into voltammetry, amperometry, potentiometry, and electrochemical impedance spectroscopy (EIS). Different voltammetric schemes are used for nitrate detection, such as cyclic voltammetry (CV), differential pulse voltammetry (DPV), square-wave voltammetry (SWV), and linear sweep voltammetry (LSV). The differences between these strategies lie in the way the voltage is applied. In CV, the current generated during nitrate reduction is determined by changing the anodic and cathodic sweep of the working electrode potential. Comparatively, LSV is the simplest method, where the applied potential is increased linearly with the corresponding current measurement over time [48]. On the other hand, in DPV and SWV, pulse techniques are used by superimposing a potential staircase. In SWV, the difference between forward and reverse pulse currents indicates the nitrate concentration. In DPV, the current is measured just before and after applying each pulse. Then, the current difference is obtained for the applied staircase potential [6]. Among the various available electrochemical methods, DPV is the most widely used technique for real-time nitrate detection, since it is suitable for field deployment, requires a short processing time, and is cost-effective [49].

**Table 1:** Advantages and disadvantages of two- and three-electrode designs.

	Advantages	Disadvantages	Ref.
Two-electrode	Suitable for portable or handheld sensors Provides a cost-effective design Simple design and implementation Easy to operate	Limited dynamic range Prone to drift	[50,51]
Three-electrode	Accurate and stable results Wider dynamic range of detection Reduced drift and improved reliability	Expensive and larger sensor size Increased complexity of measurement setup Requires regular maintenance and calibration	[52,53]

Extensive research has been conducted in the past decade utilizing IDEs to identify dissolved impurities in the form of ions in water for the purpose of water quality assessment. Techniques such as DPV, LSV, and CV are frequently employed with three-electrode sensors. Conversely, as previously mentioned, impedance and capacitance are the two measurement techniques that are often used with two-electrode interdigitated sensors (IDEs) [21] to detect the presence of ions in water or other aqueous solutions, and they are favored for their compact size, affordability, ease of fabrication, and avoidance of destroying the sample [54]. Capacitance and impedance are related to each other, with impedance being a broader term that encompasses both resistance and reactance. Reactance is further divided into two components, namely capacitive and inductive reactance. Capacitive measurements are performed by treating the material under test as a dielectric and measuring the amount of charge stored within the material. The charge in the solution builds up around the oppositely charged surface of the electrodes, and this accumulation of charge closely resembles the working principle of a capacitor [55]. This concept can be extended to nitrate ions, and variations in the density of the ionic content of the MUT would influence the dielectric properties. To analyze the capacitive behavior of IDEs, Alahi et al. [56] studied the capacitance sensitivity of IDEs with a nitrate-sensitive polymer coating on top of the electrodes, achieving a limit of detection of 4 mg/L to 14 mg/L with capacitance measurements of 5.3 nF to 5.9 nF, respectively. This approach was explored further by Ludena-Choez et al. [20], who studied the capacitance sensitivity of IDEs using graphene as the electrode material for the detection of the nitrate concentration in water. Their temperature-controlled experiment showed linearity with respect to temperature at −2.54 kΩ ${}^{\circ }$C, with a nitrate level of 1.71 ppm in agricultural soil as the limit of detection. Impedance measurement is well-suited for interdigitated sensors due to its ability to capture a broader range of electrical changes and provide more detailed information about the sensing behavior.

Bui et al. [49] reported a paper-based electrochemical sensor for the detection of both nitrate and mercury ions in lake water and polluted agricultural runoff using the DPV technique. Functionalized disposable carbon paper was used for the electrodes. Selenium particles (SePs) and gold nanoparticles (AuNPs) were synthesized to modify the electrode. The AuNPs functioned as a catalyst for the nitrate ion reduction and nucleation sites for mercury ions. The modified electrode showed high sensitivity towards nitrate and mercury without any interference. The limit of detection reported for nitrate was 8.6 μM and for mercury 1.0 ppb. Thus, this sensor showed significant promise for the simultaneous detection of nitrate and mercury ions in potable and environmental water. In another assay [22], Cu electrodes were prepared by the thermal annealing of Cu nanowires. The authors observed a favorably shaped reduction peak for even low nitrate concentrations via DPV. They reported an LOD of 1.35 μM with a sensitivity of 1.375 μA/μM at a signal-to-noise ratio of 3. Similarly, Mumtarin, Zannatul, et al. [57] reported a simple and economical approach for nitrate sensing via reduction reactions on a Cu immobilized platinum surface in a neutral medium. The sensor parameters were ascertained by the DPV technique. The sensor exhibited excellent sensitivity (2.3782 μA μM^−1^cm^−2^), a very low LOD (0.159 μM; S/N = 3), and a long-term storage ability with good reproducibility. In the same vein, a nanostructured sensor based on a copper nanowire array was obtained using the simple method of galvanic deposition. This sensor had a quick reaction time and could detect concentrations below 10 μM [58].

In amperometric methods, the current is measured over time at a constant applied potential. The current is proportional to the nitrate concentration. It has been mentioned in the literature that this method has excellent sensitivity, with low nitrate detection limits [6]. For example, Inam et al. [59] developed a nitrate sensor with a sensitivity of 19.578 μA/mM and an LOD of 0.207 nM or 0.012 μg/L using flexible screen-printed electrodes functionalized with electrodeposited copper. Furthermore, Can F. et al. [35] fabricated a biofilm electrode for amperometric nitrate measurement with a low LOD of 0.17 mM and a sensitivity of 300 nA/mM.

Potentiometry is another distinguished electrochemical nitrate detection method that is widely used for complex sample matrices like soil. It measures the potential difference between the ion-selective electrode (ISE) fabricated with a nitrate-specific ionophore and the reference electrode in the absence of a current. The ISE can be either a liquid-contact ISE (L-ISE) or a solid-contact ISE (S-ISE), based on the contact type of the inner side of the membrane. The maintenance of S-ISEs is easy compared to that of L-ISEs. Hence, S-ISEs are preferred over L-ISEs for nitrate detection, where they serve as ion-to-electron transducers [6]. EIS is another more highly sensitive electrochemical method. In EIS, the system can be characterized by measuring the sensor impedance. Generally, a sinusoidal signal with an amplitude of 5–15 mV is connected to the working electrode, and the resulting voltage is measured from the sensing electrode. The overall measurement process is carried out in a specific frequency range to obtain the impedance profile [60]. Alahi et al. [61] used an interdigital capacitive sensor functionalized by an acrylic resin with an embedded coating material for nitrate detection, and the detection limit was 1–10 mg/L, with the sensor costing less than USD 10. Similarly, Ali et al. [34] reported a microfluidic nitrate sensor using an enzyme-modified electrode with a sensitivity of 0.316 kΩ/μM/cm^2^. Even though EIS-based sensors require less sample pretreatment, the sensitivity of these sensors is lower compared to amperometric or potentiometric sensors [6]. Table 2 summarizes the specifications of different electrochemical methods.

Thus, the electrochemical analysis of nitrate detection has been explored extensively by researchers due to its short response time, inexpensive instrumentation, and applicability for direct onsite measurement [61]. Wan et al. [65,66] demonstrated a rapid and sensitive procedure to determine lead and copper simultaneously using a commercial screen-printed gold electrode modified with gold nanoparticles. A variety of catalysts including metals and nanomaterials have also been incorporated to improve the overall performance of electrochemical assays. Noteworthy advancements have been achieved to determine nitrate-specific electrochemical properties by applying various synthesis processes [6]. For example, Essousi et al. [67] developed an ion-imprinted polymer-coated working electrode with Cu nanoparticles to improve sensitivity. On the other hand, Wu et al. [68] used Cu nanoparticles and thermal oxidation to lower the LOD (12.2 μM). Even though these methods are based on hand-held devices, they still have disadvantages, like their high cost and the requirement for a person to travel to the sampling site. Additionally, it is difficult to synchronise the data obtained from several sample points. Most importantly, data are not acquired in real time, and many additional features may not be available. Thus, many of these methods are not suitable for in situ monitoring due to their complex measurement procedures. This shows the need for investigating new inline monitoring nitrate detection techniques to acquire real-time data with minimal processing costs [61,69].

## 3. Optical Methods

### 3.1. Working Principle

The principle of optical techniques is based on the behavior of a sample when exposed to electromagnetic radiation (ultraviolet, visible, or infrared). Various materials, including organic, inorganic, living, and non-living materials, are suspended and dissolved in water. When light shines on the water’s surface, a portion is reflected off the surface, while the rest travels through the water, interacting with the suspended and dissolved matter. The components of suspended and dissolved matter that can interact with electromagnetic radiation via absorption, refraction, and scattering are known as optically active constituents (OACs). Common optical characteristics that can be used to measure the concentration include the absorption or emission of radiant energy, the refraction of radiant energy, the scattering of radiant energy, and the delayed emission of radiant energy [70]. Typically, the optical instrument comprises a light source; a light detector; and various optical parts such as lenses, mirrors, prisms, and gratings. Among the various optical techniques, UV spectroscopy is widely used for nitrate detection due to its simplicity, versatility, and feasibility. Optical sensors utilize the UV absorption spectrum to directly measure nitrate in real time while in the field. These sensors’ approach of using the UV absorbance of nitrate is in line with how benchtop UV spectrophotometry is performed in a laboratory. Within these sensors, a photometer gauges the absorbance, which is the amount of light absorbed in the solution at a specific wavelength. The absorbance has a logarithmic relationship with the transmittance.

**Figure 5:**
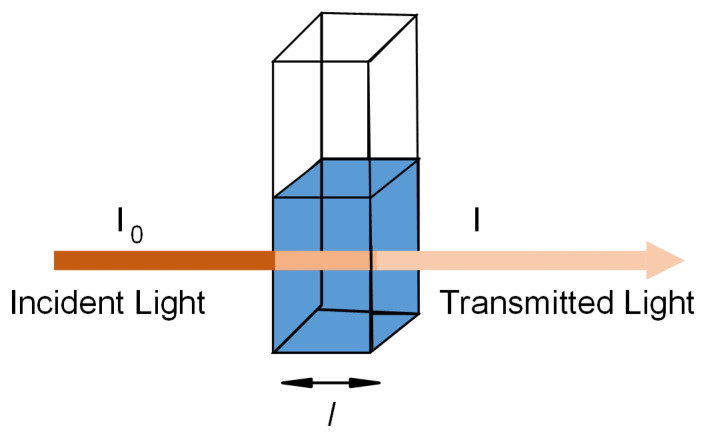
Transmission of light through a sample solution.

The transmittance, T, of the solution, is defined as the ratio of the transmitted intensity, I, over the incident intensity, I_0_, as shown in Figure 5 and represented by

(1) $$T=\frac{I}{I_{0}}$$

The absorbance, A, of the solution is related to the transmittance and incident and transmitted intensities through the following relations:

(2) $$A=log_{10}\frac{I_{0}}{I}$$

(3) $$A=-log_{10}A$$

### 3.2. Wavelength Selection

This technique for analyzing nitrate concentrations relies on measuring the absorbance at 210 nm. However, there are several interferences caused by other substances, such as chlorine, nitrite, iron (III), and organic matter, that also absorb in this region, making it difficult to accurately determine the nitrate concentration. To address this issue, the American Public Health Association (1992) developed a protocol that involves measuring the difference in sample absorbances at 220 and 275 nm using distilled water as a blank. This method helps to account for the interference caused by dissolved organic matter, which can absorb UV radiation at 220 nm, while nitrate does not absorb at 275 nm. Previous methods used for the direct UV spectroscopy of nitrate involved measuring the absorbance at 210 nm and another wavelength in the region where nitrate does not absorb UV radiation [71].

### 3.3. Measurement Methods

There are three categories of spectrophotometric methods for determining nitrate concentrations: the Griess assay, nitrosation-based spectrophotometry, and catalytic spectrophotometry [72]. Johann Peter Griess discovered the Griess reaction in 1879 as a means to detect nitrate levels in saliva through a process called diazotisation. Since then, the Griess reaction has been utilized for a significant period of time to identify bacterial infections in the urogenital tract. Bacterial reduction reduces nitrate, the primary nitrogen oxide anion present in human urine, to nitrite, which can be detected through the Griess reaction. The Griess test involves the conversion of an aromatic amine to a diazonium salt using acidified nitrite. This is then followed by a coupling reaction to produce a brightly colored azo compound. The concentration of this compound is determined spectroscopically within the range of 500–600 nm, which helps measure the amount of nitrite present [72]. Miranda et al. [73] used vanadium (III) to reduce nitrate and detect nitrate and nitrite concentrations simultaneously, employing the acidic Griess reaction. Catalan-Carrio et al. [74] developed a compact and portable device that could detect nitrite and nitrate in water samples using the multivariate analysis of color readings from the device’s sensing sites. The device incorporated both sample analysis and calibration sites. It was based on an ionogel material with an integrated Griess reaction. This hand-held device was the first of its kind. It enabled the determination of nitrite and nitrate levels in actual water samples. Beaton et al. [75] created a portable platform for measuring nitrate levels in the field that used the Griess assay and microfluidic technology. This was the first of a new generation of small analyzers, and it demonstrated low power consumption. To ensure accuracy, the system utilized on-chip absorption cells made from colored PMMA, which blocked out any background light. As a result, the system was highly sensitive and had a broad dynamic range (0.025 to 350 μM), making it suitable for analyzing nitrate levels in different types of natural water [76]. A comparative overview of different optical techniques is presented in Table 3.

Furthermore, significant advancements have been made in the development of fiber-optical sensors for analyzing water quality. These sensors comprise three crucial components: a light source, an optical fiber, and a photodetector responsible for detecting the optical signal. When selecting an optical sensor, there are two main categories to choose from: intrinsic and extrinsic sensors. Intrinsic sensors rely on a continuous and consistent light source to enable a phase-modulation technique. This modulation takes place within the optical fiber, and the sensor design specifically employs a single-mode fiber. On the other hand, extrinsic optical sensors utilize the fiber as a means of transmitting the optical signal. They are particularly advantageous for remote sensing applications due to their compact size and low power requirements. Additionally, extrinsic sensors find utility in the detection of nitrate and nitrite levels in water [3]. Camas-Anzueto et al. [77] reported an extrinsic sensor with lophine as a sensitive layer on the fiber. The sensor could detect concentrations of 1 to 70 mg/L and had a response time of 20 milliseconds. The sensor operated at a wavelength range of 300–1100 nm for detection. In a similar manner, Chong and colleagues utilized fiber optics and optical sensing technology to investigate nitrate levels in water. They reported wavelengths of 350 nm to 2500 nm to detect nitrate, and the detection range was 0–2.50 mg/L. To illuminate the samples, they utilized a halogen lamp in conjunction with an ASD FieldSpec 3 Hi-Res Portable Spectro-radiometer. A disposable optical sensor was created for detecting nitrate. The sensor utilized a recognition system founded on an artificial C_3_ symmetry amide-based ionophore and had the ability to detect nitrate within a broad range of concentrations (26 μMM to 63 mM). However, this sensor has been noted to have longer response times of up to 5 min and a reduced accuracy as its drawbacks [50].

Sensors that use spectrophotometers and do not need reagents have shown potential for monitoring nitrate in the environment. Nevertheless, additional research is necessary to tackle sensor drift, which can occur over extended periods. It is worth highlighting that the deuterium light sources found in UV/Vis spectrophotometric systems are highly stable and can last up to 5000 h (around 7 months). However, there are significant issues with chemical fouling, which causes chemical precipitation, and biofouling, which leads to the accumulation of organic substances on the sensor optics. As a result, the regular cleaning and rinsing of the sensors are required when measuring nitrate [78].

## 4. Real-Time Nitrate Sensors and Recent Developments

In the past decade, researchers have explored different methods to develop a real-time nitrate monitoring system. Su et al. [79] reported a novel approach for the real-time monitoring of nitrate concentrations through the competitive relationship between the microbial denitrification and electrogenesis processes. This development allowed the extension of the application of bio-electrochemical technology to water technology. Bluett et al. [80] implemented an in situ ion chromatography analyzer to measure nitrate and nitrite concentrations in remote surface water applications. They also developed a self-cleaning 3D-printed sediment trap to filter the samples before the measurement, preventing silt and sediment from causing blockages. In addition, they also developed a solar-powered battery and an internal heater to facilitate off-grid deployments of the analyzer in remote locations. Beaton et al. [75] developed a lab-on-chip (LOC) colorimetric system with a membrane sample filter for measuring nitrate and nitrite levels at a remote glacier where high sediment loads were regular. They used microfluidic technology to obtain a miniaturized version of the field analyzer. The reported LOD of the system was 0.025 μM and 0.02 μM for nitrate and nitrite, respectively. On the other hand, field-effect transistor (FET)-based sensors have drawn considerable attention in chemical and biomaterial sensing due to their high sensitivity, real-time response, outstanding performance, straightforward operation, and label-free detection. Chen et al. [81] reported an rGO-based FET platform modified with benzyl triethylammonium chloride (TEBAC) for the sensitive and selective electronic detection of nitrate ions in water. This sensor could detect nitrates from 0.0028 to 28 mg/L and was suitable for real-time and in situ application.

Current alternative methods include inline analysis with electronic instruments. In recent years, the use of machine learning for estimating water quality through remote sensing has gained popularity due to advancements in algorithm development, sensor systems, computing power, and data accessibility. Machine learning refers to a collection of statistical techniques that allow a computer to automatically learn from data and develop models for detection, estimation, or classification, minimizing the difference between the training and prediction datasets without explicit programming. This approach, also known as statistical learning, involves feeding data into a computer that can be “trained” using predefined features or objects to enable semi-automated or automated detection, classification, or pattern recognition [70]. Additionally, wireless sensor networks (WSNs) and Internet of Things (IoT) technologies have been rigorously investigated for inline water quality monitoring in the literature [82,83,84,85,86]. WSNs expand the abilities of in situ monitoring systems. Although classic in situ approaches allow computation on site, they require that the gathered information be transported manually to control centers or remote stations for further examination and action. WSNs allow the automatic transfer of data. They also provide a feedback process in some instances to enhance the quality of data collection. Generally, a WSN includes a sensor unit, referred to as the sensing nodes; a processor for signal processing; interfacing circuitry; a transmitter/receiver system for connectivity; and a power supply [87]. WSNs have obvious advantages such as wireless communication, multiple inline sample points, temporal and spatial evolution, a low cost, and fast deployment [88]. The strengths and weaknesses of various wireless transmission protocols used in WSNs are summarized in Table 4.

Various authors have reported WSN-based water quality monitoring systems in the past few years. For example, Gartia et al. [91] designed miniature nitrate sensors to monitor nitrate concentrations through a WSN. This method had the constraint that it was a sample-based system and unable to provide continuous inline measurements. Corke et al. [85] developed a sensor node to monitor salinity in ground waters as well as the water temperature in surface waters. Tuna et al. [92] proposed portable sensor nodes mounted on buoys with wireless interfaces to monitor electrical conductivity, dissolved oxygen, pH, temperature, turbidity, and nitrate. Wang et al. [93] presented a wireless network based on TinyOS, LabVIEW, and MySQL for pH, nitrate, and phosphate monitoring. However, due to the multiple sensor nodes, energy-harvesting techniques and hibernation played a crucial role in extending the system’s lifespan [86]. Nasser et al. [94] implemented a WSN-based water quality monitoring system using Squidbee sensor motes and pH sensors to provide pH data in real time. They designed a system with an information portal and an alternate sleep mechanism to prolong the network lifetime. In [69,95], the researchers developed a WSN and a solar-panel-based energy harvesting system to detect nitrate, chloride, and ammonium concentrations in lakes and rivers.

The IoT allows any physical entity to be sensed and monitored and to transmit data to a specific server via the Internet. Thus, it makes it possible to integrate the physical world into computer-based systems, which enhances precision and decreases human intervention. Extensive computing, smart sensors, embedded devices, communication technologies, Internet protocols, and applications are essential for the IoT. Recently, the IoT has been developed and applied in water quality monitoring. A smart system that uses IoT technology to analyze sensor data can automatically monitor water quality and promptly alert water analysts if there are any abnormalities. The implementation of machine-to-machine communication makes it easier and more efficient to analyze and communicate data [96]. IoT-enabled WSNs assist in securing more entity connections for monitoring purposes and help in building smart cities, smart industries, smart agriculture, etc.

In [60], the authors integrated a WSN with the IoT by designing a smart sensing node to monitor nitrate levels in the field. The sensing node collected water from a lake, stream, or river; instantly measured the nitrate concentration; and transferred the data through the gateway to a user-defined cloud server. Agir et al. [97] proposed an electro-analytical wireless sensor for the online monitoring of nitrate and ammonium in water. They developed ion-selective nitrate and ammonium electrodes and combined them with a portable IoT system. Similarly, in another study [98], the authors proposed a real-time water quality monitoring system based on a wireless sensor network with IoT capabilities. This affordable system could perform the real-time analysis of various water quality parameters such as pH level, temperature, nitrate, chloride, and dissolved oxygen, issuing timely warnings. Yet another IoT-enabled real-time smart nitrate sensing system was developed in [99], where the authors reported the fabrication process of carbon nanotubes (CNTs) with PDMS polymer coupled with graphene-based interdigitated sensors.

## 5. Discussion

Conventionally, nitrate level measurement is accurately performed offline in the laboratory. The disadvantages of such offline methods have already been discussed in the Introduction. Additionally, it is understood that when samples are stored, their quality may be compromised by various factors, such as biological activity and matrix effects, despite adherence to proper handling protocols [69]. Inline analysis has significant benefits over traditional offline methods. Firstly, it eliminates or reduces sample contamination resulting from sample handling, as there is minimal sample transformation involved. Secondly, it reduces the overall cost of data acquisition by saving time on sampling and the handling and analysis of the sample [69].

**Figure 6:**
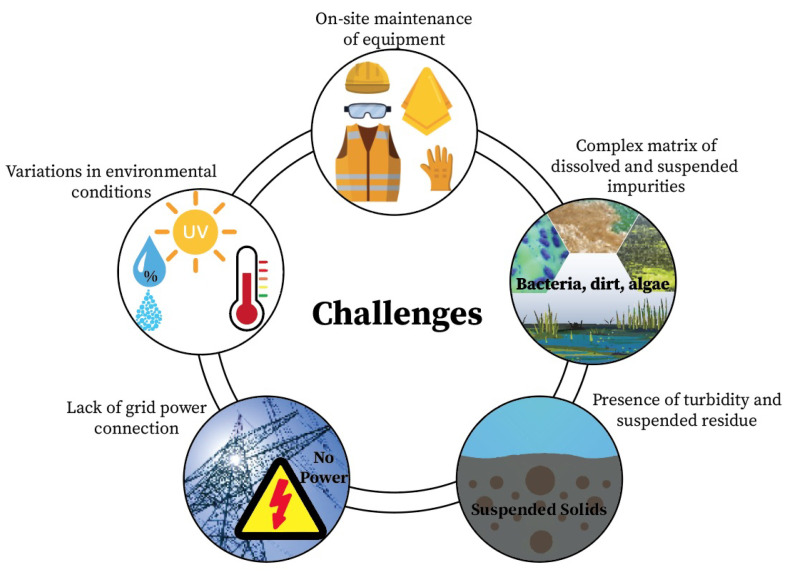
Challenges during implementing real-time nitrate sensors.

Several analytical techniques can be conceivably employed for the real-time detection of nitrate, such as chromatography, UV spectroscopy, various colorimetric methods, and electrochemistry [80]. However, each of these methods encounters one or more challenges during implementation (Figure 6). Most of the time, natural waters are situated in remote places, where there can be a lack of grid-power connection. Environmental and temperature conditions are not constant all the time. Additionally, factors such as changing levels of turbidity and suspended residue in natural waters can limit the performance reliability of the analytical equipment [16,80]. Furthermore, restrictions on accessing, cleaning, and servicing should be considered. Many inline nitrate sensors require the regular and effective cleaning of the probes to ensure accurate readings. The cost of cleaning is a significant factor, sometimes making up 50% of the operational expenses. Common cleaning techniques include ultrasonic, brush, water-jet, and chemical-based automatic cleaners. However, some new and cost-effective methods have been suggested, such as electrolysis, which actively removes buildup, or using copper mesh or CuO_2_-doped materials, which passively prevent fouling [84].

The techniques discussed in this review that can be incorporated in autonomous field-deployed applications are a step towards the fourth industrial revolution, often referred to as Industry 4.0 [100]. The continuous monitoring of contaminants in water is beneficial and useful for environmental agencies and city councils. Currently, water quality monitoring largely takes place in laboratories, where trained personnel operate expensive machinery in specialized facilities. Sample collection also represents an arduous process, which involves careful transportation to the lab. Industry 4.0 factors in the cost and labor involved and offers low-cost solutions to monitor the quality of water by researching techniques that can be implemented using sensitive and selective sensors characterized by a small size, making them applicable for the autonomous monitoring of nitrate ions in water.

There is a strong correlation between suspended residue levels and turbidity. In UV-absorbance-based optical nitrate analyzers, turbidity induces a scattering effect that influences the absorption spectrum and results in detection errors. Furthermore, many dissolved components are present in natural water that absorb the UV wavelength and influence the UV sensor response due to interference and matrix effects [80,101].

Colorimetric analyzers offer potential for real-time nitrate determination due to the lower energy demand and reagent consumption of microfluidic systems [102]. Nevertheless, colorimetry is susceptible to temperature changes and necessitates constant calibration using reference standards to compensate for these alterations [16]. Additionally, sample turbidity variations can influence analysis accuracy [103]. The colorimetric reagents and standards required are often hazardous and toxic, making the development and upkeep of remote systems more difficult.

The popularity of using electrochemical analysis to detect nitrates is increasing due to its advantages, including the fast response times, affordable equipment, and ability to perform measurements on site. However, current research on this type of analysis has certain limitations, such as the need for sample collection, challenges in synchronizing data from multiple sample points, and the inability to obtain real-time data or additional features. As a result, existing electrochemical methods are not suitable for on-site monitoring due to their complex measurement procedures. Table 5 presents a summary of the strengths and weaknesses of different approaches that could be utilized to install nitrate sensors in real time. Nevertheless, there is potential for electrochemical analysis to be used for real-time nitrate detection.

## 6. Conclusions

The utilization of thin-film electrodes for the detection of nitrate ions in water has shown great potential for accurate and efficient analysis. The unique geometric design with closely spaced fingers provides a large surface area for enhanced interaction with nitrate ions. This feature enables the sensitive detection and precise quantification of nitrate levels, making interdigitated planar electrodes a valuable tool for water quality monitoring. Various detection techniques have been employed with interdigitated planar electrodes to detect nitrate ions in water, including impedance spectroscopy, cyclic voltammetry, and amperometric methods. Each technique offers distinct advantages, such as high sensitivity, rapid responses, and a wide dynamic range. Researchers have been able to customize detection techniques based on their specific requirements and optimize the performance of interdigitated planar electrodes for nitrate ion detection.

As research in this field continues to advance, further improvements in interdigitated planar electrode design, material selection, and detection techniques can be explored. Such improvements have shown enhanced sensitivity, selectivity, portability, and reliability, which are ideal for applications that require field deployment. A few studies have also explored real-time systems, determining the amount of nitrate ions in water while providing sensor data instantaneously. This involves interfacing the sensing electrodes with an IoT platform to enable the remote monitoring of sensor data.

In this review, we delved into the wide range of detection techniques, including various field-deployable electrochemical and optical methods such as optical fibers and UV–visible spectroscopy, that can be used to monitor nitrate levels in real time, but primarily focusing on electrochemical methods that have been integrated with planar electrodes to achieve the targeted detection of nitrate ions in water. The review comprehensively examined the advantages and limitations of various detection techniques, as well as the utilization of both two-electrode and three-electrode systems. Furthermore, we provided an overview of the existing research conducted on real-time nitrate monitoring systems. Interdigitated electrodes have gained popularity in the past two decades for the detection of nitrate ions in conjunction with impedance and capacitive measurement techniques. Among these techniques, impedance measurements have gained popularity due to their simplified measurement process, which can also be achieved using dedicated impedance converter integrated circuits (ICs). We discussed the intricacies and advancements of impedance-based measurement methods and highlighted their widespread use in nitrate ion detection with interdigitated electrodes. By examining these diverse detection techniques and their integration with planar electrodes, this review shed light on the progress made thus far in the field of nitrate ion detection in water. The findings contribute to a better understanding of the strengths and limitations of various approaches, paving the way for further advancements in real-time nitrate monitoring systems and the improvement of water quality management strategies.

Looking ahead, the field of nitrate ion detection in water using interdigitated planar electrodes holds promising prospects for future advancements. One potential direction for future research is the development of miniaturized and portable detection systems that can allow the real-time monitoring of nitrate levels in various water sources. This would enable the rapid and in situ assessment of water quality, facilitating proactive measures to address nitrate pollution and ensure the safety of drinking water. Furthermore, there is scope for exploring novel materials and surface modifications for interdigitated electrodes to enhance their sensitivity and selectivity towards nitrate ions. The integration of nanoparticles or conductive polymers could offer enhanced electrochemical properties and facilitate more precise nitrate ion detection. It is also possible to combine planar electrodes in schemes such as microwave resonators [104,105,106,107]. Furthermore, the integration of 3D-printed microfluidics [108] could allow systems to work more efficiently. Additionally, the incorporation of smart sensing technologies, such as wireless communications and data analysis algorithms, could enable the development of intelligent nitrate monitoring systems that could perhaps be linked to weather forecasts to predict rises in nitrate levels and inform authorities.

Sensors that measure various physical parameters are often subjected to harsh environments, which makes building robust sensors challenging. There is a high demand for properties such as repeatability, sensitivity, low cost, and selectivity in sensors, which presents opportunities for advancements in sensor technology. Several commercial sensors and systems are available, specifically designed for sensing various parameters related to water, such as water quality, nutrient levels, and heavy metals [109,110,111]. Some of these systems can even provide real-time monitoring by utilizing sensors for continuous data collection. However, it is important to note that each system comes with its own set of advantages and disadvantages that should be carefully considered during the selection process.

## Figures and Tables

**Table 2:** Comparison of various electrochemical methods.

Method	Electrode	LOD	Linear Range	Sensitivity	R^2^	Ref.
DPV	PEG-SH/SeP/AuNP-modified carbon paper	8.6 μM				[49]
DPV	Cu nanowires	1.35 μM	8 to 5860 μM	1.375 μA/μM	0.999	[22]
DPV	Cu-immobilized platinum surface	0.159 μM	0.12 to 4.75 mM	2.3782 μA/μM		[57]
LSV	Cu nanowire array	9.1 μM	10 to 50	0.0636		[58]
			50 to 1500 μM	0.73 μA/μM		
Amperometry	Screen-printed silver	0.207 nM	0.05 to 5 mM	19.578 μA/μM	0.987	[59]
Amperometry	Screen-printed graphite	100 nM	0.1 to 10 mM	0.12 μA/μM	0.999	[62]
Amperometry	Screen-printed carbon		0.01 to 0.25 nM	3.13 μA/μM	0.97	[63]
Amperometry	Polypyrrole/carbon nanotube film	0.17 mM	0.44 to 1.45 mM	300	0.97 nA/mM	[35]
Potentiometry	Screen-printed carbon	100 mM	0.1 to 100 mM			[64]
EIS	Interdigital capacitive	1 to 10 mg/L				[61]
EIS	Graphene foam-based		1 to 1000 μM	0.316 kΩ/μM/cm^2^		[34]

**Table 3:** Comparison of various optical methods.

Method	Absorbance Range	LOD	Dynamic Range	Sensitivity	Ref.
Spectrophotometry	540 nm	3 μM	3–334 μM	0.5 μM	[73]
UV-Vis spectrometry	365 nm	2.3 mg/L	2.3–3.4 mg/L		[74]
Colorimetry		0.025 μM	0.025–350 μM	0.025 μM	[75]
Fiber optic	575 mn		0–2.5 mg/L		[76]

**Table 4:** Comparison of wireless transmission protocols.

Wireless	Merits	Shortcomings	Ref.
Transmission			
Bluetooth	1. Simple and convenient	1. Less distance covered	[89]
	communication	2. Supports fewer nodes	
	2. Data transfer rate can meet	3. Transmission stability	
	the requirements for short-distance transmission	is not high	
	3. Completely free	4. Unreliable communication	
Wi-Fi	1. High bandwidth	1. Short range	[18]
	2. Fast communication due	2. High power consumption	
	to higher transmission speed	3. Cost is involved	
	3. A variety of large-scale	4. Security issues	
	wireless device manufacturers		
	are confirmed		
ZigBee	1. Low power consumption	1. Low data speed	[86]
	2. Easy to install	2. Low transmission, as well as	
	3. Supports a large number of nodes	low network stability	
	4. High-reliability network		
	5. Strong security		
LoRa	1. Low power consumption	1. Limited bandwidth	[47]
	2. Long-range coverage	2. Limited message size	
	3. Supports a large number of nodes	3. Complex network setup	
Sigfox	1. Low power consumption	1. Limited bandwidth	[90]
	2. Long-range coverage	2. Limited network availability	
	3. Low cost	3. Small data packet	
	4. Easily scalable		

**Table 5:** Summary of potential methods.

Potential Methods	Strengths	Limitations
Colorimetry	Lower energy consumption Use of microfluidic system reduces reagent quantity	Influenced by temperature variation Needs regulator calibration Requires regular cleaning of sensor Reagents are often hazardous and toxic Turbidity variations affect accuracy
UV spectroscopy	High sensitivity Non-destructive technique Rapid measurements Wide wavelength range	Sensitive to turbidity Prone to inaccurate data due to sample matrix Variability in absorbance maxima
Electrochemistry	Quick response Affordable equipment Wide dynamic range Non-destructive technique Ability to perform on-site measurements	Suffers from drift overtime Sensitivity to temperature changes Prone to inaccurate data due to sample matrix

## Data Availability

Data sharing not applicable.
